# Host Factors Associated with Gut Mycobiome Structure

**DOI:** 10.1128/msystems.00986-22

**Published:** 2023-02-14

**Authors:** Natalia Szóstak, Luiza Handschuh, Anna Samelak-Czajka, Katarzyna Tomela, Marcin Schmidt, Łukasz Pruss, Kaja Milanowska-Zabel, Piotr Kozlowski, Anna Philips

**Affiliations:** a Institute of Bioorganic Chemistry, Polish Academy of Sciences, Poznan, Poland; b Institute of Computing Science, Poznan University of Technology, Poznan, Poland; c Department of Cancer Immunology, Chair of Medical Biotechnology, Poznan University of Medical Sciences, Poznan, Poland; d Department of Food Biotechnology and Microbiology, Poznan University of Life Sciences, Poznan, Poland; e Ardigen S.A., Cracow, Poland; f Department of Biochemistry, Molecular Biology and Biotechnology, Faculty of Chemistry, Wroclaw University of Science and Technology, Wroclaw, Poland; Cleveland Clinic

**Keywords:** gut mycobiome, intestinal microbiota, high-throughput sequencing, taxonomy profiling, bioinformatics, next-generation sequencing

## Abstract

Recent studies revealed a significant role of the gut fungal community in human health. Here, we investigated the content and variation of gut mycobiota among subjects from the European population. We explored the interplay between gut fungi and various host-related sociodemographic, lifestyle, health, and dietary factors. The study included 923 participants. Fecal DNA samples were analyzed by whole-metagenome high-throughput sequencing. Subsequently, fungi taxonomic profiles were determined and accompanied by computational and statistical analyses of the association with 53 host-related factors. Fungal communities were characterized by a high prevalence of *Saccharomyces*, *Candida*, and *Sporisorium*. Ten factors were found to correlate significantly with the overall mycobiota variation. Most were diet related, including the consumption of chips, meat, sodas, sweetening, processed food, and alcohol, followed by age and marital status. Differences in α- and/or β-diversity were also reported for other factors such as body mass index (BMI), job type, autoimmunological diseases, and probiotics. Differential abundance analysis revealed fungal species that exhibited different patterns of changes under specific conditions. The human gut mycobiota is dominated by yeast, including *Saccharomyces*, *Malassezia*, and *Candida*. Although intervolunteer variability was high, several fungal species persisted across most samples, which may be evidence that a core gut mycobiota exists. Moreover, we showed that host-related factors such as diet, age, and marital status influence the variability of gut mycobiota. To our knowledge, this is the first large and comprehensive study of the European cohort in terms of gut mycobiota associations with such an extensive and differentiated host-related set of factors.

**IMPORTANCE** The human gut is inhabited by many organisms, including bacteria and fungi, that may affect human health. However, research on human gut mycobiome is still rare. Moreover, the large European-based cohort study is missing. Here, we analyzed the first large European cohort in terms of gut mycobiota associations with a differentiated host-related set of factors. Our results showed that chips, meat, sodas, sweetening, processed food, beer, alcohol consumption, age, and marital status were associated with the variability of gut mycobiota. Moreover, our analysis revealed changes in abundances at the fungal species level for many investigated factors. Our results can suggest potentially valuable paths for further, narrowly focused research on gut mycobiome and its impact on human health. In the coming era of gut microbiome-based precision medicine, further research into the relationship between different mycobial structures and host-related factors may result in new preventive approaches or therapeutic procedures.

## INTRODUCTION

In recent years, microbiomes of various environments, including different human body sites, have attracted much attention from the scientific community. One of the most intriguing habitats is the human gut, which is populated by many microorganisms belonging to multiple kingdoms. Consecutive studies slowly reveal a bidirectional relationship between intestinal microorganisms and human health. So far, most efforts have been put into the investigation of the ecology and diversity of bacterial communities. Meanwhile, recent studies have revealed a substantial role of the human gut fungal community, known as the mycobiota, in the gut ecosystem and human health (1–3).

Several factors have been suggested to be associated with alterations in mycobiota community composition, such as host genetics, sex, age, comorbidities, drugs, lifestyle, socioeconomic status, diet, occupation, and the immune system. However, despite the growing interest in the gut mycobiome, this area of research is still in its infancy. For example, a core mycobiota has not been determined yet (1), and the consensus as to whether fungi form a long-term, stable community in the human gut has still to be reached.

Until today, the associations between host-related factors and human intestinal mycobiota have mainly been investigated in Chinese cohorts (4), with a notable exception of the research devoted to the analysis of the Human Microbiome Project, where 317 samples from healthy donors were characterized in terms of gut fungi (5). Other studies on the human gut mycobiome were narrow and mostly concentrated on a specific condition. Such as research on the gut mycobiome in relation to diet (6), age and gender (7), body mass index (BMI) (8), specific diseases, e.g., liver diseases (see references 9 and 10 for reviews), irritable bowel disease (11, 12), autoimmunological conditions (13–15), and antibiotics (16–19).

Meanwhile, when analyzing the associations between microbiota and host-related factors, one has to take into account that factors may vary considerably depending on the geographic region and culture, and so do the environment and genetics across different populations. Thus, the results of gut mycobiota multiomic analyses and the conclusions drawn from them should be considered in the context of sociodemography. As Chinese and Eastern diet, lifestyle, and environmental factors vary significantly from European ones, the results and conclusions drawn from the research on the relationship between gut mycobiota and diet based on Chinese cohorts may not apply to other populations. Therefore, to understand the connection between gut mycobiota and different factors, it is crucial to perform analyses on different cohorts.

In this study, we performed whole-metagenome sequencing and profiled gut fungal community structure in a Central European cohort (*n* = 923). We have also accompanied our analysis with a comprehensive assessment of gut mycobiota covariates. In parallel to fecal sampling, we have collected participant metadata such as anthropometric and lifestyle data, eating habits, and medications (a total of 53 variables). To estimate the impact of different factors that may influence the gut mycobiome structure, we ran a wide range of statistical tests that allowed us to describe a variation of the fungal communities in the context of the host-related factors and identify differentially abundant species.

## RESULTS

### Gut mycobiota variation.

We characterized gut fungal communities in 923 fecal samples gathered from volunteers from Poland. Each participant completed a detailed medical, lifestyle, and dietary questionnaire (see Table S1 in the supplemental material).

10.1128/msystems.00986-22.1 TABLE S1 Participant data analyzed during the study. Download Table S1, XLSX file, 0.3 MB. Copyright © 2023 Szóstak et al. 2023 Szóstak et al. https://creativecommons.org/licenses/by/4.0/ This content is distributed under the terms of the Creative Commons Attribution 4.0 International license.

**FIG 1:**
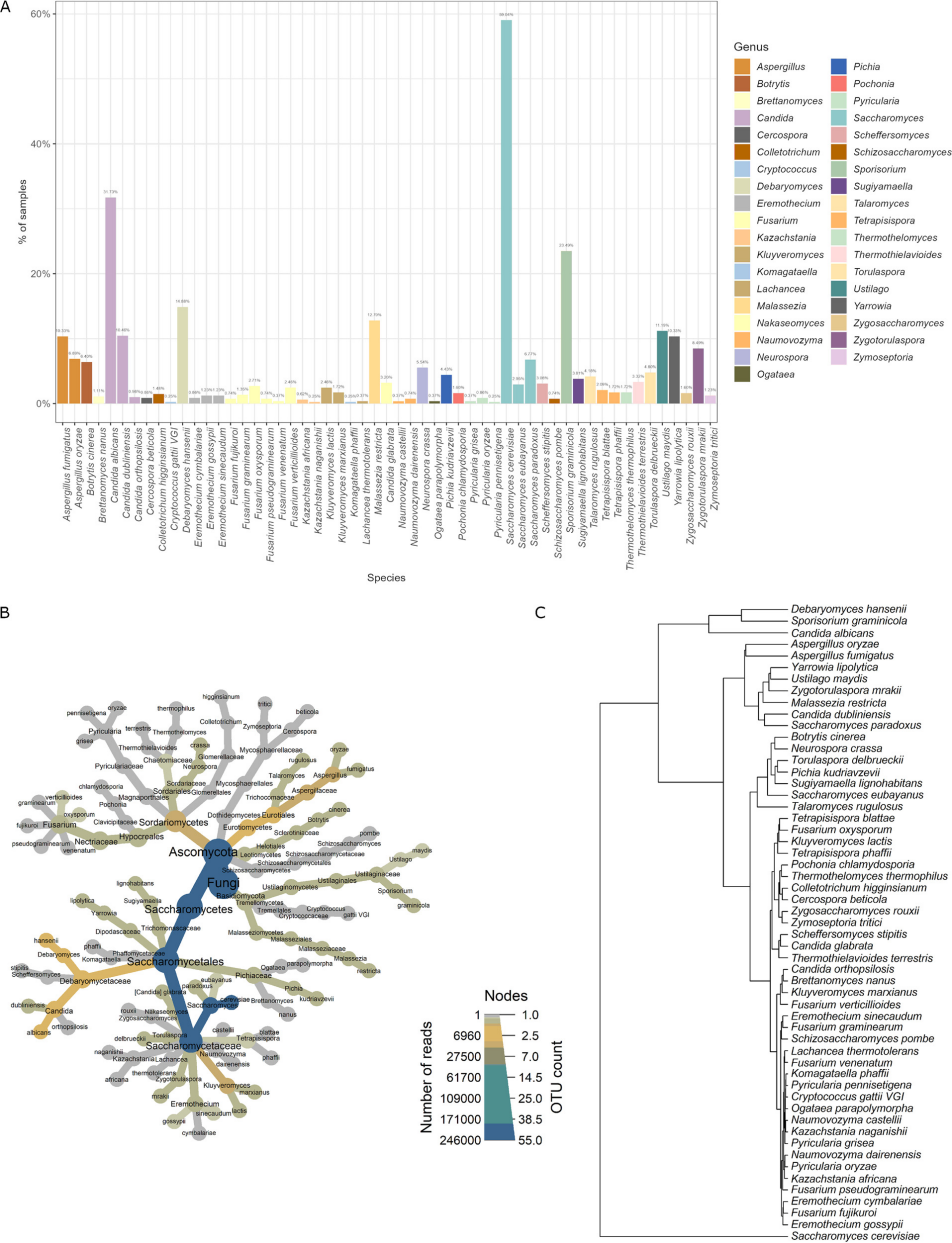
Variation of fungal mycobiota in the investigated samples. (A) Overall prevalence of fungal species in the investigated fecal samples. The percentage represents the number of samples in which a given species was identified. (B) Overall mycobiome structure of investigated gut samples. The size of nodes is set according to the number of different operational taxonomic units (OTUs) at a given taxonomical level. Color is intensified according to the number of reads for a given OTU. (C) Dendrogram of fungal species based on their frequency in the samples.

To characterize the fungal lineages present in the fecal microbiota, we extracted DNA from stool and performed whole-metagenome high-throughput sequencing (next-generation sequencing [NGS]) with NextSeq 550. Fungi were detected in 88.1% of subjects (813 samples), and these samples were further analyzed. In total, 55 different species were detected in the samples, belonging to 37 genera, 20 families, 12 orders, 9 classes, and 2 phyla (Table S2). *Ascomycota* was the dominant phylum (present in 94.0% of the samples), while *Basidiomycota* was present in 40.0%. The most prevalent genus was *Saccharomyces* (present in 59.0% of the samples), followed by *Candida* (present in 38.7% of the samples) and *Sporisorium* (present in 23.4% of the samples). At the species level, the most prevalent species was Saccharomyces cerevisiae (present in 58.8% of the samples), followed by Candida albicans and Sporisorium graminicola (present in 31.6% and 23.4% of the samples, respectively) (Fig. 1A). The overall mycobiome structure of the investigated samples is presented in Fig. 1B. The fungal richness of individual stool samples was low, as an individual sample contained, on average, 2.77 (standard deviation [SD], 1.82) different fungal species, and the maximum number of species identified in one sample was 11.

10.1128/msystems.00986-22.2 TABLE S2 Fungal taxonomical units identified in the samples. Download Table S2, XLSX file, 0.02 MB. Copyright © 2023 Szóstak et al. 2023 Szóstak et al. https://creativecommons.org/licenses/by/4.0/ This content is distributed under the terms of the Creative Commons Attribution 4.0 International license.

Clustering of the fungal species co-occurrences shows that some species more often coexist with others (Fig. 1C). As a dominant fungal species in human gut microbiota (present in 58.79% of samples), S. cerevisiae was clustered separately. One cluster is created by C. albicans, Debaryomyces hansenii, and S. graminicola, which are most commonly found in analyzed samples (31.61%, 23.37%, and 14.76% of analyzed samples, respectively). Together with S. cerevisiae, these fungi may appear as the core fungal microbiota in the human gut, although all of them, except S. cerevisiae, are found in less than 50% of individuals. Aspergillus fumigatus, Talaromyces rugulosus, Aspergillus oryzae, Saccharomyces paradoxus, Candida dubliniensis, Malassezia restricta, Zygotorulaspora mrakii, Ustilago maydis, and Yarrowia lipolytica constitute another distinct cluster. In this cluster, fungi found with medium frequency in investigated samples can be seen (average frequency of 8.91%; maximum and minimum frequency of samples, 12.55% and 4.06%, respectively). Clustering also shows three other groups, mainly fungi found in the tested samples with a lower frequency.

**FIG 2:**
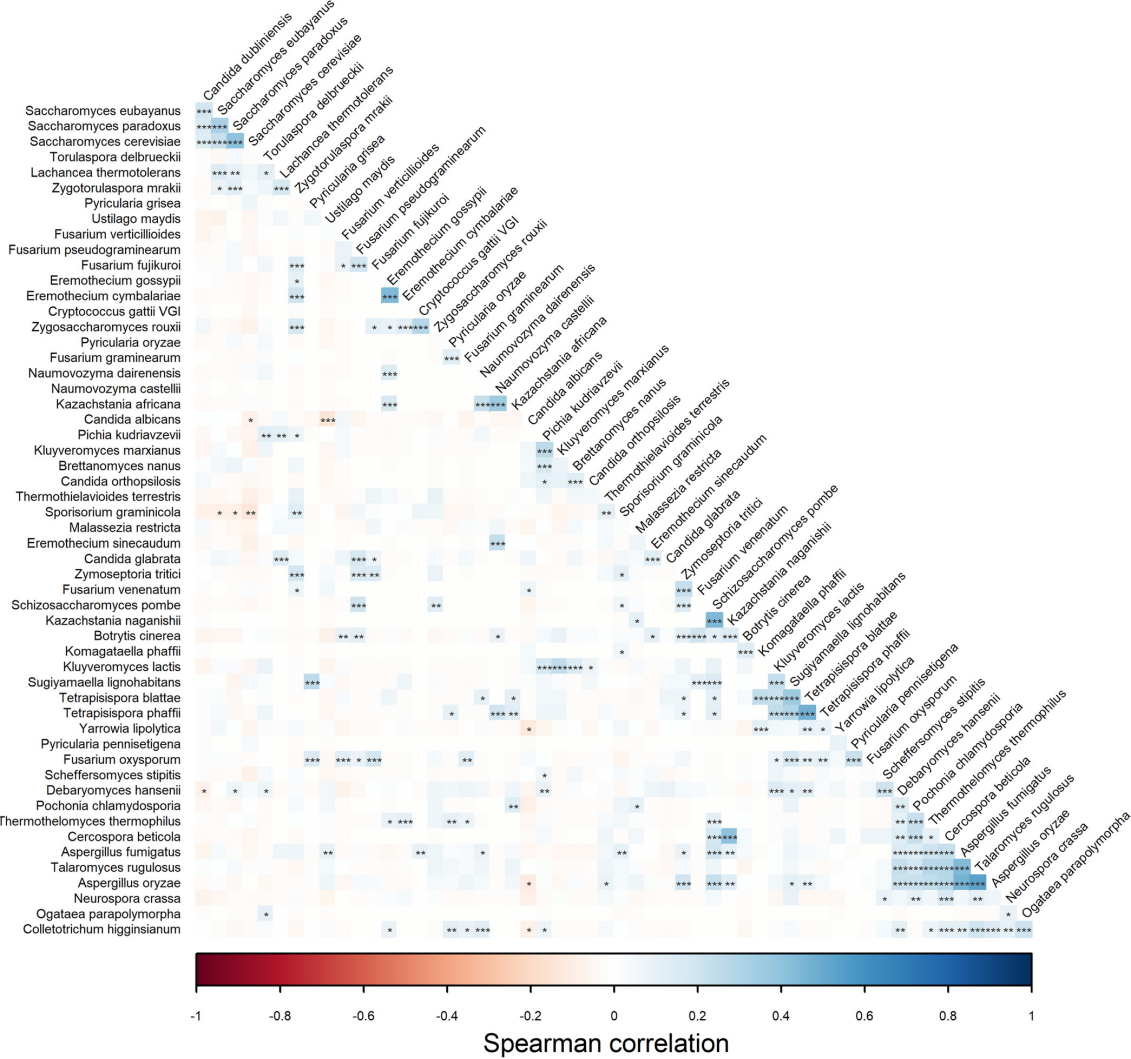
Associations between species. Pairwise correlations were calculated with Spearman correlation, and their significance was estimated with the *t* test with FDR adjustment; *P* = 0.05 was used for confidence intervals. Two-tailed probability of *t* test for each correlation was used as an indicator of significance. Significant associations are marked as follows: ***, *P* = 0.001; **, *P* = 0.01; and *, *P* = 0.05. Color is intensified according to the correlation coefficient.

Analysis of correlations between different fungal species shows a number of significantly associated species (Fig. 2). Most of them are positively correlated, and only several are negatively correlated. Of interest, the strongest statistically significant positive correlations can be observed within the *Saccharomyces* genus, between species belonging to *Saccharomyces* and C. dubliniensis and between T. rugulosus and D. hansenii, Pochonia chlamydosporia, Thermothelomyces thermophilus, Cercospora beticola, and species belonging to Aspergillus. The same correlations can be observed for A. fumigatus and A. oryzae. Significant mild negative correlations were detected between C. albicans and U. maydis, S. cerevisiae and Colletotrichum higginsianum, and between S. graminicola, Saccharomyces eubayanus, and S. paradoxus.

### Host factors associated with gut mycobiome composition.

**FIG 3:**
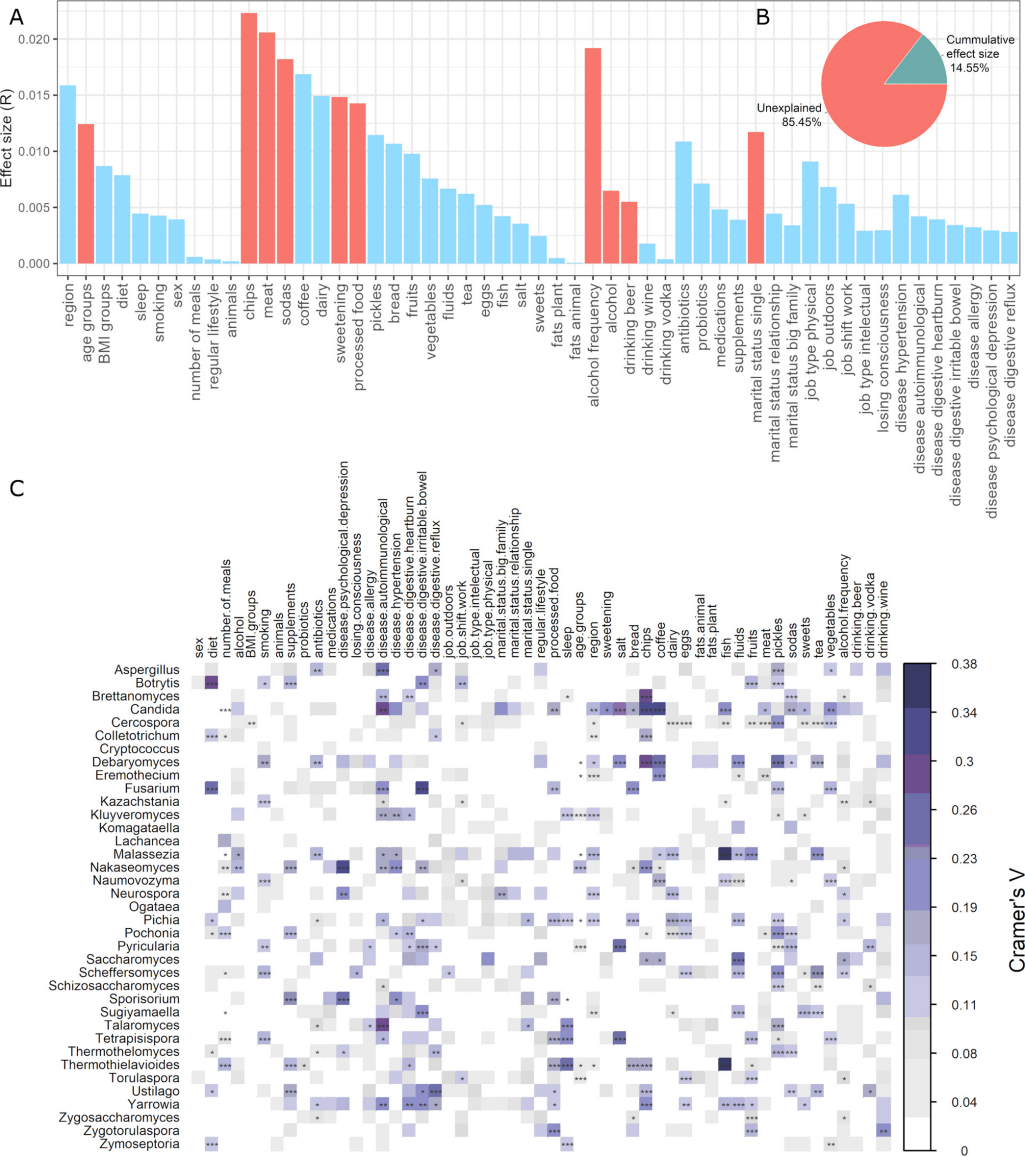
Host factors and their effect size on gut mycobiota variation. (A) Effect size of participant data variables in human gut mycobiota variation. Mycobiota covariates were identified via envfit (vegan), and those with statistical significance (FDR-adjusted *P* < 0.05) are colored red. (B) Pie chart showing the fraction of microbial variation explained by all statistically significant human data variables. (C) Associations between gut mycobiota and investigated factors. Correlations coefficients were calculated with Cramer V, and their significance was estimated with the chi-square test. Significant associations are marked as follows: ***, *P* = 0.001; **, *P* = 0.01; and *, *P* = 0.05. Color is intensified according to the correlation coefficient. The data were log-ratio centered (clr transformation) before investigation.

To identify factors that are significantly associated with gut mycobiome composition, we tested 53 participant data variables. Before the examination, data were log-ratio centered (clr transformation). Ten factors were found to correlate (*P *< 0.05) with the overall mycobiota variation of the analyzed population (Fig. 3A). Among them, diet-related factors, including consumption of chips, meat, and sodas and alcohol consumption frequency, were the top four covariates of the gut mycobiota. They were followed by the usage of sugar (sweetening), consumption of processed food, and age groups. Interestingly, being single was also detected as significantly associated with gut mycobiota variation among the tested population. The last two factors contributing to the observed mycobiota variation were alcohol usage and drinking beer. Altogether, all these statistically significant participant data variables explained 14.54% of the mycobiota variation (Fig. 3B). Neither investigated job-related factors nor antibiotics, probiotics, supplements, and medications were significantly associated with gut mycobiome composition (*P *> 0.05). The same insignificant associations were attributed to analyzed diseases.

We have also performed a correlation analysis between fungal genera and investigated human data. Again, data were clr transformed prior to the correlation analysis. The analysis revealed that a wide range of fungal genera were mildly associated with different factors, with a prevalence of disease and diet-related factors (*P *< 0.05) (Fig. 3C). Patterns of association vary highly with respect to a specific factor. Several statistically significant moderate associations can be observed for depression (*Nakaseomyces*, *Neurospora*, *Sporisorium*), autoimmunological disorders (Aspergillus, *Candida*, Fusarium, *Talaromyces*, *Yarrowia*), hypertension (*Nakaseomyces*, *Sporisorium*), heartburn (*Ustilago*), irritable bowel (*Botrytis*, Fusarium), and reflux (*Ustilago*). The diet factor is moderately correlated with *Botrytis* and Fusarium. Among diet-related factors, also moderate correlations can be observed for sweetening (*Candida*), salt (*Candida*, *Debaryomyces*, *Pyricularia*, *Tetrapisispora*), chips (*Brettanomyces*, *Candida*, *Colletotrichum*, *Debaryomyces*, *Nakaseomyces*, *Thermothielavioides*, *Yarrowia*), coffee (*Candida*, *Debaryomyces*, *Eremothecium*, *Naumovozyma*), fish (*Candida*, *Malassezia*, *Thermothielavioides*), fluids (*Debaryomyces*, *Saccharomyces*), pickles (*Cercospora*, *Debaryomyces*, *Pochonia*, *Scheffersomyces*, *Talaromyces*), tea (*Debaryomyces*, *Malassezia*, *Scheffersomyces*), vegetables (*Candida*), and drinking wine (*Zygotorulaspora*). Interestingly, sleep was correlated with a few fungal genera, *Talaromyces*, *Tetrapisispora*, and *Thermothielavioides*.

### The effect of diet on gut mycobiota.

**FIG 4:**
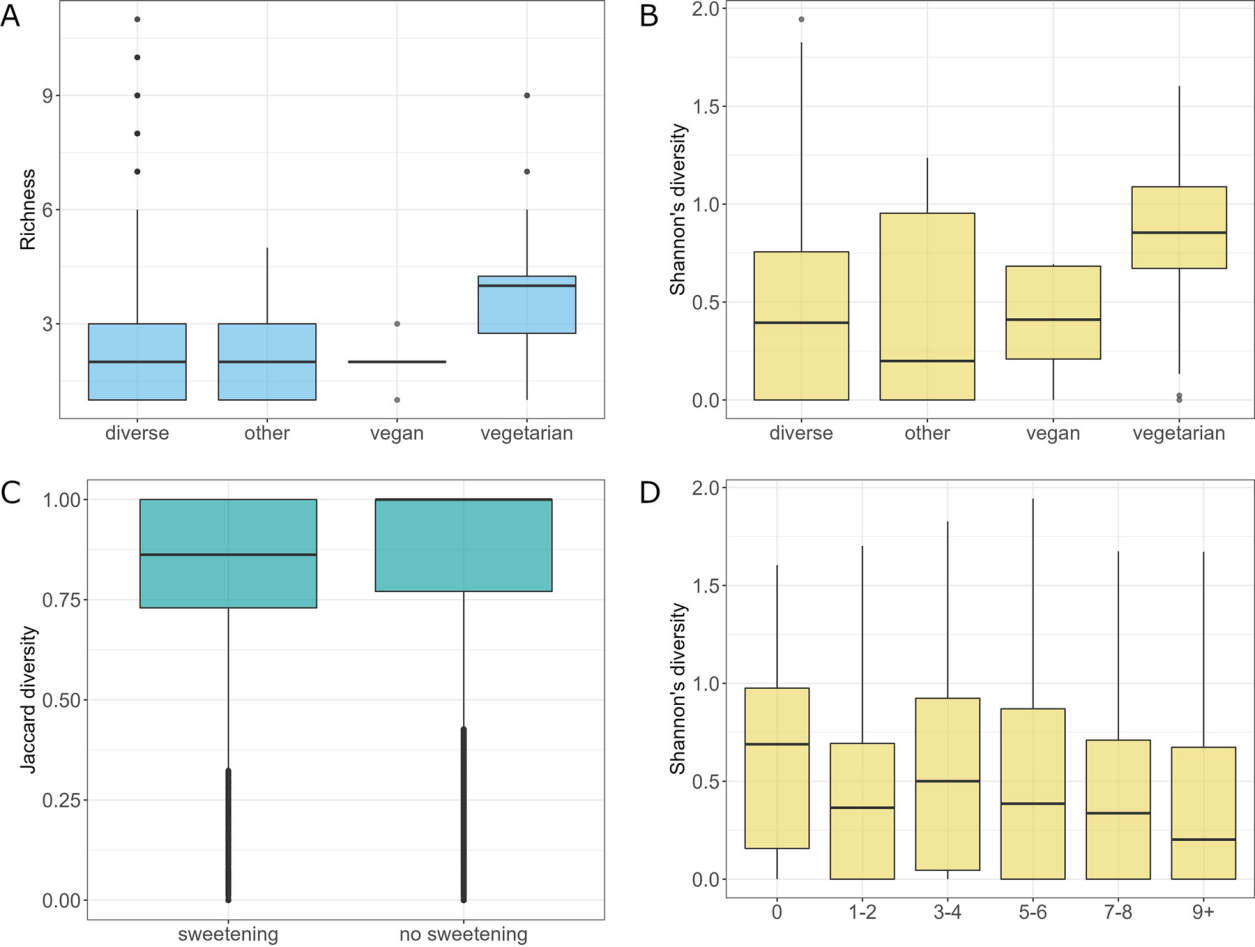
Characteristics of mycobiota in different dietary conditions. (A) Richness of the analyzed community with respect to the diet type. (B) Shannon diversity of mycobiota concerning diet type. (C) β-Diversity for the sweetening factor (clr-transformed data). (D) Shannon diversity for the meat consumption factor.

To study the influence of the diet-related factors on the host mycobiota, 795 samples that have appropriate participant data regarding the diet were divided into 4 groups reflecting the general diet type of the subjects, diverse, other, vegan, and vegetarian (*n* = 730, *n* = 28, *n* = 5, and *n* = 32, respectively). Statistical analysis showed differences in fungal species richness between tested groups (Kruskal-Wallis rank-sum test, *P *< 0.001). Pairwise comparison using Conover's nonparametric test allowed for a detailed comparison and showed that vegetarians had significantly higher gut fungal richness than subjects declaring diverse or other type of diet (*P *< 0.05) (Fig. 4A). Similarly, analysis of Shannon diversity revealed the differences between diet groups (Kruskal-Wallis rank-sum test, *P *< 0.001). Vegetarians had significantly higher Shannon diversity than subjects declaring diverse or other type of diet (*P *< 0.05 for comparisons with diverse and other type of diet groups, pairwise comparison using Conover's nonparametric test [Fig. 4B]). Differential abundance analysis of mycobiota revealed many fungal species that differ in abundance in subjects with a specific type of diet (analysis of compositions of microbiomes with bias correction [ANCOM-BC], *P *< 0.05) (Fig. S1).

10.1128/msystems.00986-22.4 FIG S1 Waterfall plot for the diet factor. (A) Vegetarian versus diverse diet. (B) Vegan versus diverse diet. (C) Other types of diet versus diverse diet. (D) Vegan versus vegetarian diet. (E) Vegetarian versus other types of diet. (F) Vegan versus other types of diet. Only statistically significant fungal species are illustrated (ANCOM-BC, *P *< 0.05). Download FIG S1, TIF file, 0.6 MB. Copyright © 2023 Szóstak et al. 2023 Szóstak et al. https://creativecommons.org/licenses/by/4.0/ This content is distributed under the terms of the Creative Commons Attribution 4.0 International license.

Compared to a diverse type of diet, vegetarians had a significantly higher abundance of A. oryzae and S. paradoxus and a lower abundance of Neurospora crassa (Fig. S1A). Vegans had a significantly higher abundance of Botrytis cinerea, Y. lipolytica, U. maydis, S. graminicola, A. oryzae, S. paradoxus, N. crassa, and M. restricta and a lower abundance of Z. mrakii, A. fumigatus, C. dubliniensis, D. hansenii, and S. cerevisiae (Fig. S1B). Subjects with an “other” type of diet had a significantly higher abundance of B. cinerea, N. crassa, Y. lipolytica, and A. oryzae and a lower abundance of S. paradoxus, M. restricta, Z. mrakii, S. graminicola, and C. dubliniensis (Fig. S1C). A comparison of vegans versus vegetarians revealed that differences were partially similar to differences between vegans and subjects with a diverse type of diet. Vegans had a higher abundance of B. cinerea, S. graminicola, U. maydis, and Y. lipolytica and a lower abundance of D. hansenii, M. restricta, A. fumigatus, C. dubliniensis, C. albicans, and S. cerevisiae (Fig. S1D). In comparison between vegans and subjects declaring an “other” type of diet, vegans had a slightly higher abundance of U. maydis and C. albicans and a lower abundance of A. fumigatus, D. hansenii, and S. cerevisiae (Fig. S1F). Vegetarians had a higher abundance of Y. lipolytica, S. graminicola, and B. cinerea and a lower abundance of C. dubliniensis, M. restricta, and Z. mrakii (Fig. S1E).

For the sweetening factor, the analysis did not reveal statistically significant differences in richness and Shannon and Pilou diversities between the group of subjects who sweetened and not sweetened (true versus false in the sweetening category). However, analysis of variance within different sweetening behaviors revealed statistically significant differences in β-diversity between tested groups (adonis, clr-transformed data, *P *= 0.016, *R*^2^ = 0.002) (Fig. 4C). Differential abundance analysis revealed that C. dubliniensis and S. paradoxus were the species that were more abundant in the group of subjects that sweetened (ANCOM-BC, *P *< 0.05) (Fig. S2).

10.1128/msystems.00986-22.5 FIG S2 Waterfall plot for the sweetening effect. Only statistically significant fungal species are illustrated (ANCOM-BC, *P *< 0.05), and no sweetening was treated as a reference. Download FIG S2, TIF file, 0.1 MB. Copyright © 2023 Szóstak et al. 2023 Szóstak et al. https://creativecommons.org/licenses/by/4.0/ This content is distributed under the terms of the Creative Commons Attribution 4.0 International license.

Fungal Shannon diversity was significantly higher in subjects not consuming meat than in subjects declaring meat consumption between one to two portions weekly (one portion equals 150 g), seven to eight portions weekly, and above nine portions weekly (Conover's nonparametric all-pairs comparison test with continuity correction, *P *< 0.05) (Fig. 4D). Differential abundance analysis of mycobiota revealed two fungal species that differ in abundance in subjects with different consumption of meat (ANCOM-BC, *P *< 0.05) (Fig. S3). For almost all groups declaring consumption of meat above zero, N. crassa and S. paradoxus had higher abundance than in subjects who did not consume meat, except for the group declaring consumption of meat at the level of three to four portions, which had a positive log fold change for N. crassa but negative log fold change for S. paradoxus, although the negative log fold change was relatively small.

10.1128/msystems.00986-22.6 FIG S3 Waterfall plot for the meat consumption factor. (A) Subjects declaring weekly consumption of meat above nine portions, compared to subjects declaring no meat consumption (9+ versus 0). (B) Subjects declaring weekly consumption of meat at the level of seven to eight portions compared to subjects declaring no meat consumption (7 to 8 versus 0). (C) Subjects declaring weekly consumption of meat at the level of five and six portions compared to subjects declaring no consumption of meat (5 to 6 versus 0). (D) Subjects declaring weekly consumption of meat at the level of three to four portions compared to subjects declaring no consumption of meat (3 to 4 versus 0). (E) Subjects declaring weekly consumption of meat at the level of one to two portions compared to subjects declaring no meat consumption (1 to 2 versus 0). One portion of meat equals 150 g. Only statistically significant fungal species are illustrated (ANCOM-BC, *P *< 0.05). Download FIG S3, TIF file, 0.4 MB. Copyright © 2023 Szóstak et al. 2023 Szóstak et al. https://creativecommons.org/licenses/by/4.0/ This content is distributed under the terms of the Creative Commons Attribution 4.0 International license.

Despite their significant association with gut mycobiome composition, analysis of chips, sodas, and processed food factors did not reveal statistically significant differences in richness, Shannon diversity, Pilou diversity, and β-diversity between the tested groups within all the human data analyzed.

Subjects with daily consumption of sodas above 2 L had a significantly higher abundance of S. cerevisiae than subjects declaring no consumption of sodas and a slightly lower abundance of B. cinerea, Y. lipolytica, N. crassa, and A. oryzae (ANCOM-BC, *P *< 0.05, log fold change > 0.1) (Fig. S4A). The log fold change for S. cerevisiae was relatively high, exceeding the value of 1. Interestingly, comparing subjects with consumption of sodas in a range between 1.5 and 2.0 L to subjects declaring no consumption of sodas, we observed that A. oryzae content had positive log fold change, while the negative changes could be observed for similar species as in the comparison of subjects with consumption of sodas above 2.0 L and no consumption of sodas, with the only difference being Z. mrakii that replaced A. oryzae (ANCOM-BC, *P *< 0.05, log fold change > 0.1) (Fig. S4B). With the decrease in sodas consumption to 1.0 to 1.5 L daily, subjects had a higher abundance of C. dubliniensis, S. paradoxus, and A. oryzae and a lower abundance of B. cinerea, M. restricta, N. crassa, and Z. mrakii (ANCOM-BC, *P *< 0.05, log fold change > 0.1) (Fig. S4A).

10.1128/msystems.00986-22.7 FIG S4 Waterfall plot for the soda consumption factor. (A) Subjects declaring daily consumption of sodas above 2.0 L compared to subjects declaring no consumption of sodas (2.0+ versus 0). (B) Subjects declaring daily consumption of sodas between 1.5 L and 2.0 L compared to subjects declaring no consumption of sodas (1.5 to 2.0 versus 0). (C) Subjects declaring daily consumption of sodas between 1.0 l and 1.5 L, compared to subjects declaring no consumption of sodas (1.0 to 1.5 versus 0). Only statistically significant fungal species are illustrated (ANCOM-BC, *P *< 0.05). Download FIG S4, TIF file, 0.3 MB. Copyright © 2023 Szóstak et al. 2023 Szóstak et al. https://creativecommons.org/licenses/by/4.0/ This content is distributed under the terms of the Creative Commons Attribution 4.0 International license.

In the case of chip consumption, subjects who declared weekly consumption of chips above five portions (one portion equals 30 g) had a higher abundance of S. paradoxus, C. dubliniensis, U. maydis, M. restricta, and Y. lipolytica and a lower abundance of Z. mrakii, N. crassa, B. cinerea, A. oryzae, D. hansenii, A. fumigatus, and S. graminicola than subjects with no consumption of chips (ANCOM-BC, *P *< 0.05, log fold change > 0.1) (Fig. S5A). The differences disappear with the decrease in consumption of chips, and subjects declaring weekly consumption of chips at the level of three to four portions differ from the group of zero consumption only by the decline of one fungal species, Z. mrakii (ANCOM-BC, *P *< 0.05, log fold change > 0.1) (Fig. S5B).

10.1128/msystems.00986-22.8 FIG S5 Waterfall plot for chip consumption factor. (A) Subjects declaring weekly consumption of chips above five portions compared to subjects declaring no consumption of chips (5+ versus 0). (B) Subjects declaring monthly consumption of chips at the level of three to four portions compared to subjects declaring no consumption of chips (3 to 4 versus 0). One portion of chips equals 30 g. Only statistically significant fungal species are illustrated (ANCOM-BC, *P *< 0.05). Download FIG S5, TIF file, 0.2 MB. Copyright © 2023 Szóstak et al. 2023 Szóstak et al. https://creativecommons.org/licenses/by/4.0/ This content is distributed under the terms of the Creative Commons Attribution 4.0 International license.

Only frequent (a group declaring “several times a week”) processed food consumption affected the structure of the gut mycobiome. Subjects who consume processed food several times a week had a significantly higher abundance of N. crassa and a lower abundance of A. oryzae (Fig. S6).

10.1128/msystems.00986-22.9 FIG S6 Waterfall plot for the processed food factor. Only statistically significant fungal species are illustrated (ANCOM-BC, *P *< 0.05). Download FIG S6, TIF file, 2.0 MB. Copyright © 2023 Szóstak et al. 2023 Szóstak et al. https://creativecommons.org/licenses/by/4.0/ This content is distributed under the terms of the Creative Commons Attribution 4.0 International license.

### The effect of alcohol consumption on gut mycobiota.

**FIG 5:**
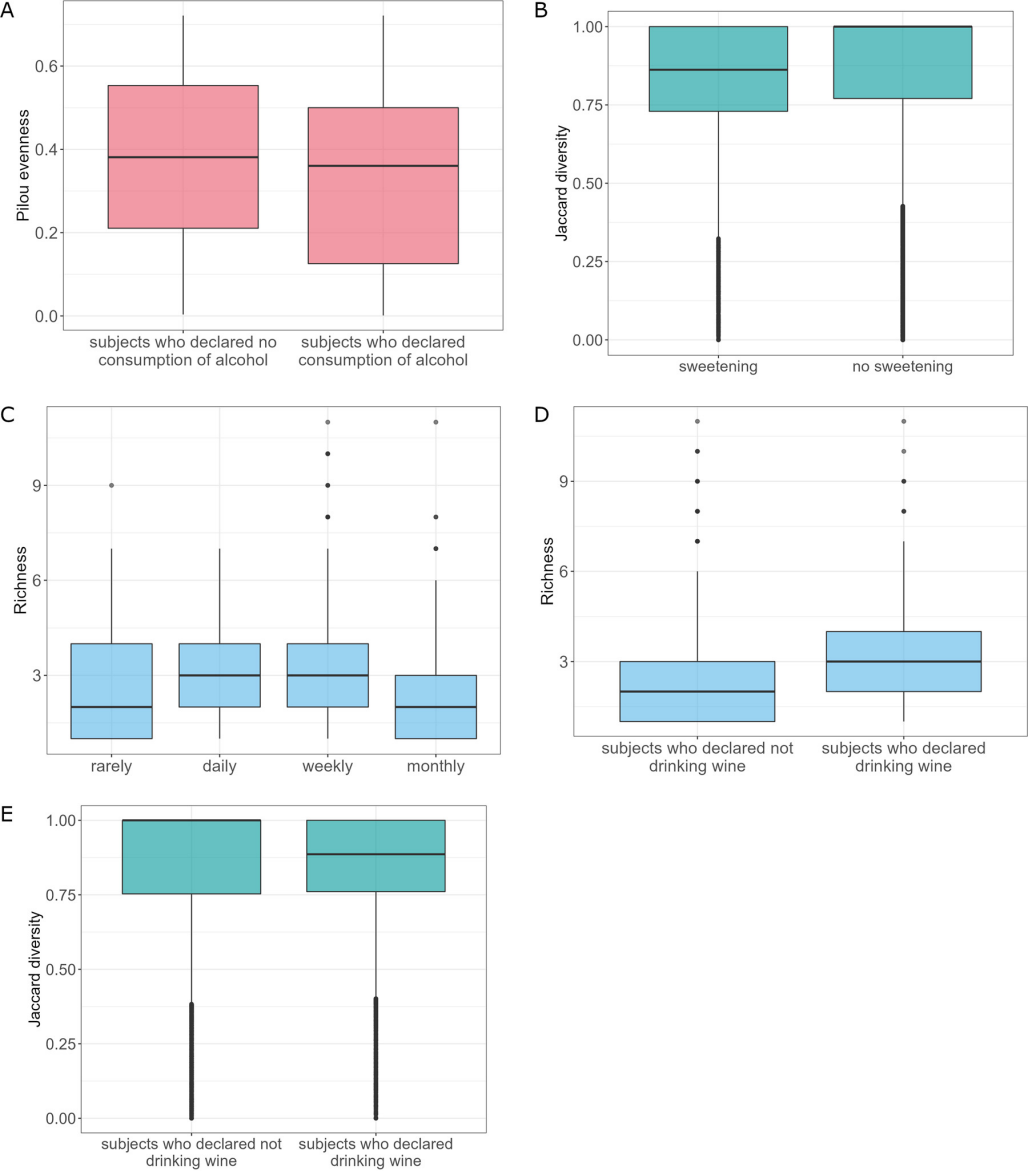
Characteristics of mycobiota taking into account different alcohol conditions. (A) Pilou evenness of the analyzed community with respect to the alcohol consumption factor. (B) β-Diversity of the analyzed community with respect to the alcohol consumption factor (clr-transformed data). (C) Richness of the analyzed community with respect to the alcohol consumption frequency. (D) Richness of the analyzed community with respect to the drinking wine factor. (E) β-Diversity of the analyzed community with respect to the drinking wine factor (clr-transformed data).

Alcohol consumption was another factor that was significantly associated with gut mycobiome composition. Alcohol factor analysis revealed statistically significant differences in terms of Pilou diversity between a group of subjects who declared drinking alcohol versus not drinking alcohol (Kruskal-Wallis rank-sum test, *P *= 0.036). Subjects who drank alcohol had lower diversified fungal microbiota (Fig. 5A). Also, analysis of variance revealed statistically significant differences in β-diversity between subjects who drink alcohol and abstinents (adonis, clr-transformed data, *P *= 0.011, *R*^2^ = 0.003) (Fig. 5B). Moreover, subjects who drank alcohol, in general, had a significantly higher abundance of S. cerevisiae than subjects declaring no consumption of alcohol (ANCOM-BC) (Fig. S7). Of notice, this may be the direct effect of alcohol consumption, as S. cerevisiae is widely known to be involved in the beer and winemaking process.

10.1128/msystems.00986-22.10 FIG S7 Waterfall plot for the alcohol consumption factor for subjects declaring consumption of alcohol compared to subjects declaring no consumption of alcohol. Only statistically significant fungal species are illustrated (ANCOM-BC, *P *< 0.05). Zero consumption was treated as a reference. Download FIG S7, TIF file, 0.1 MB. Copyright © 2023 Szóstak et al. 2023 Szóstak et al. https://creativecommons.org/licenses/by/4.0/ This content is distributed under the terms of the Creative Commons Attribution 4.0 International license.

Analysis of alcohol consumption frequency showed differences in richness between different declared alcohol consumption schemas (rarely, daily, monthly, weekly, Kruskal-Wallis rank-sum test, *P *= 0.007). Subjects declaring daily and weekly frequency of alcohol consumption had significantly higher fungal species richness than subjects who drink alcohol with monthly frequency (Conover’s nonparametric all-pairs comparison test, *P *= 0.048 and *P *= 0.033, respectively) (Fig. 5C). Interestingly, only daily consumption of alcohol affected gut mycobiota structure (Fig. S8 at https://figshare.com/articles/figure/Supplementary_Figure_8_tiff/21710075/2). Subjects with frequent (daily) consumption of alcohol had a significantly higher abundance of Z. mrakii and A. fumigatus and decreased amounts of B. cinerea, M. restricta, Y. lipolytica, U. maydis, A. fumigatus, and S. graminicola compared to subjects with rare consumption of alcohol and monthly consumption of alcohol.

Across tested alcohol types (wine, beer, and vodka), only consumption of wine revealed statistically significant differences in the fungal species richness between tested groups (Kruskal-Wallis rank-sum test, *P *= 0.006) (Fig. 5D). β-Diversity between subjects who drink wine versus subjects declaring no consumption of wine was also significant (adonis, clr-transformed data, *P *= 0.022, *R*^2^ = 0.003) (Fig. 5E). However, differential abundance analysis of mycobiota could not reveal species that differ between tested groups of subjects for none of the analyzed alcohol types.

### The effect of age and host’s sex on gut mycobiota.

**FIG 6:**
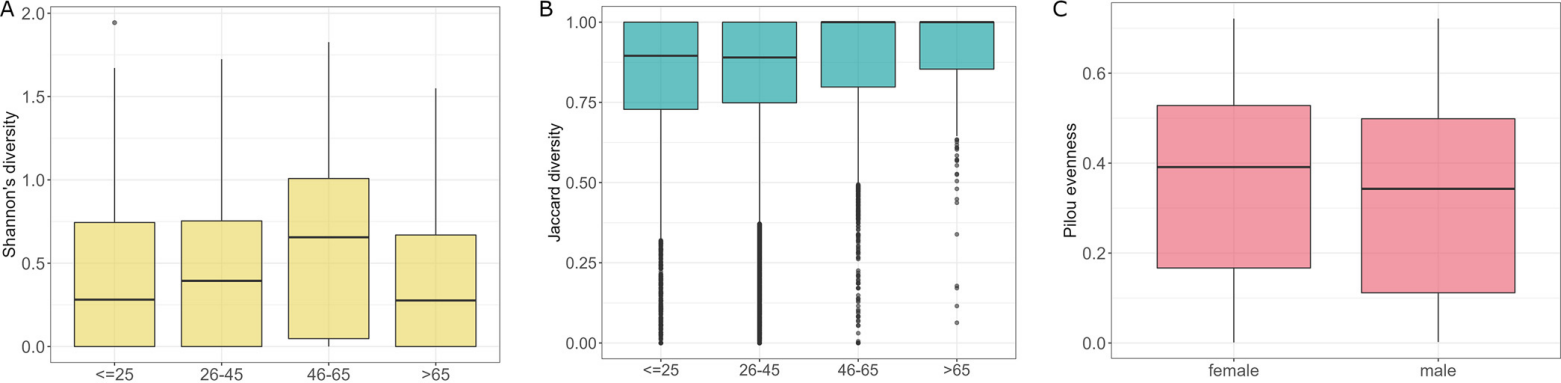
Characteristics of mycobiota, taking into account different age groups. (A) Shannon diversities of the analyzed community in different age groups. (B) β-Diversities of the analyzed community with respect to the age groups (clr-transformed data). (C) Evenness of the analyzed community with respect to the hosts’ sex.

We divided 806 samples into four groups based on the age of the subjects, ≤25, 26 to 45, 46 to 65, and >65 (*n* = 183, *n* = 484, *n* = 110, and *n* = 30, respectively). Analysis showed differences in Shannon diversity between tested groups (Kruskal-Wallis rank-sum test, *P *= 0.036). Conover’s nonparametric all-pairs comparison test allowed for more detailed comparison and showed that Shannon diversity for group 46 to 65 was significantly different from the ≤25 and 26 to 45 groups (*P *= 0.038 and *P *= 0.038): average Shannon diversity in group 46 to 65 was significantly higher (Fig. 6A). Groups differed also in β-diversity (adonis, clr-transformed data, *P *= 0.001, *R*^2^ = 0.008), with significant differences for groups 26 to 45 versus 46 to 65 and ≤25 versus 46 to 65 (pairwise adonis, adjusted *P *= 0.012 and *R*^2^ = 0.005 and adjusted *P *= 0.012 and *R*^2^ = 0.011, respectively) (Table 1; Fig. 6B).

**TABLE 1:** PERMANOVA results for age groups

Pair	F model	*R* ^2^	*P* value	*P* adjusted
26–45 vs ≤25	1.090	0.002	0.320	1.000
26–45 vs 46–65	2.752	0.005	0.002	0.012
26–45 vs >65	2.233	0.004	0.012	0.072
≤25 vs 46–65	3.276	0.011	0.002	0.012
≤25 vs >65	2.297	0.011	0.011	0.066
46–65 vs >65	1.467	0.011	0.104	0.624

Differential analysis showed that a group 46 to 65 differed from group ≤25 in the abundance of S. graminicola (positive log fold change) and S. paradoxus (negative log fold change) (Fig. S9A at https://figshare.com/articles/figure/Supplementary_Figure_9/21710108/1). Furthermore, a lower abundance of S. paradoxus was also detected in the case of comparison of group 46 to 65 to group 26 to 45, accompanied by a lower abundance of S. cerevisiae (Fig. S9B at https://figshare.com/articles/figure/Supplementary_Figure_9/21710108/1). Although Conover's nonparametric all-pairs comparison test did not reveal any statistically significant differences for other age groups’ comparisons, differential analysis of mycobiota revealed species that differ in abundance also between other age groups. The highest differences could be observed in comparison with subjects above 65 years old. Group 26 to 45 had a higher abundance of Z. mrakii, A. fumigatus, Y. lipolytica, C. dubliniensis, A. oryzae, B. cinerea, and N. crassa than group >65 (Fig. S9C at https://figshare.com/articles/figure/Supplementary_Figure_9/21710108/1). Differences in fungal species between subjects under 25 and above 65 were similar, except for A. oryzae and B. cinerea, which had negative log fold change, accompanied by S. paradoxus (Fig. S9D at https://figshare.com/articles/figure/Supplementary_Figure_9/21710108/1). Almost the same pattern of differences could be observed for group 26 to 45 in comparison with subjects above 65 years old, with the only exception of N. crassa, which also had negative fold change values in this case (Fig. S9E at https://figshare.com/articles/figure/Supplementary_Figure_9/21710108/1).

Regarding hosts’ sex, the male microbial community turned out to be less homogenous (or even) than the female microbial community, as revealed by the comparison of Pilou diversities (*P *= 0.021, Kruskal-Wallis rank-sum test) (Fig. 6C).

### The effect of BMI on gut mycobiota.

**FIG 7:**
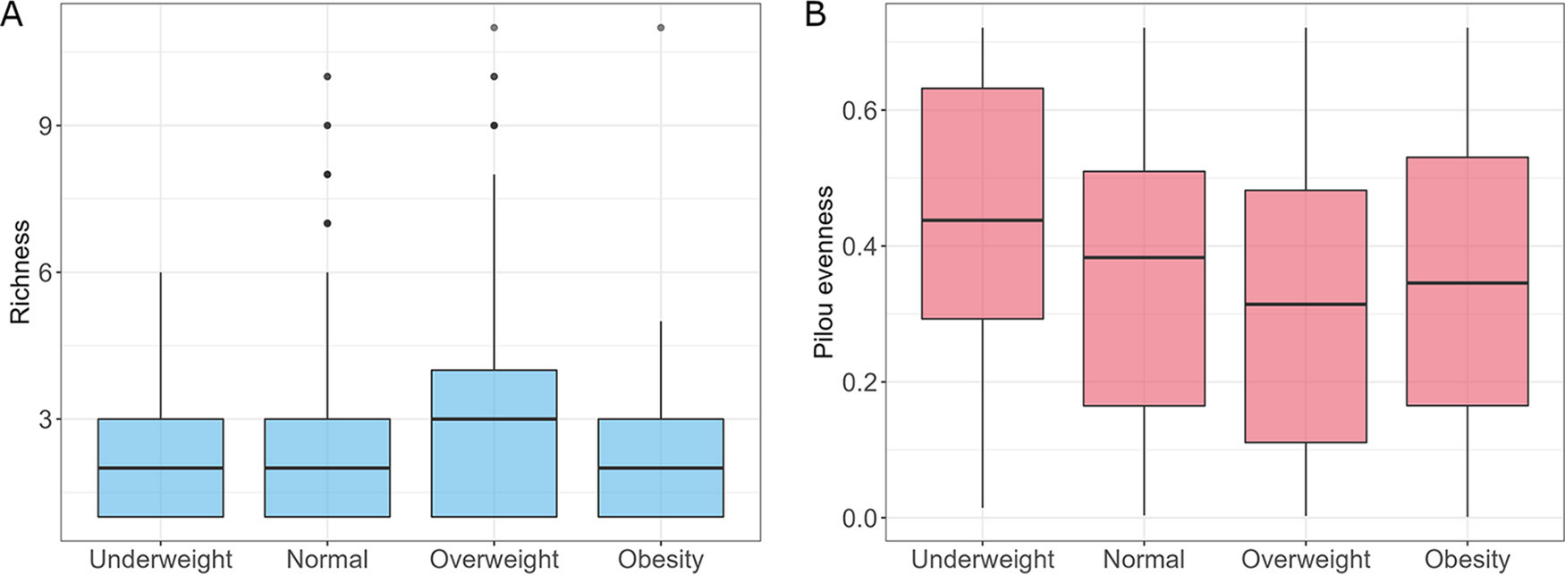
Characteristics of mycobiota, taking into account different BMI groups. (A) Richness of the analyzed community with respect to the BMI. (B) Evenness of mycobiota with respect to the BMI group.

BMI was calculated for 756 subjects based on the provided height and weight (only subjects who provided weight and height were taken into account). Next, samples were divided into four groups based on the BMI of the subjects, including underweight (BMI < 18.5, *n* = 41), normal (18.5 ≤ BMI < 25, *n* = 422), overweight (25 ≤ BMI < 30, *n* = 233), and obese (BMI ≥ 30, *n* = 61). Statistical analysis showed differences in fungal species richness between tested groups (Kruskal-Wallis rank-sum test, *P *= 0.0346) (Fig. 7A). However, a detailed pairwise comparison using the Conover’s nonparametric all-pairs comparison test did not reveal statistically significant differences in richness between tested groups. Also, fungal Shannon diversity was not significantly different between the tested groups. In contrast, Pilou evenness metrics for BMI groups differed significantly (Kruskal-Wallis rank-sum test, *P *= 0.013), and overweight subjects had considerably less homogenous mycobiota than underweight and normal subjects (Conover’s nonparametric all-pairs comparison test, *P *= 0.029 and *P *= 0.041, respectively) (Fig. 7B).

Differential analysis revealed a diversified pattern of changes in fungal species between tested groups. Underweight subjects had a higher abundance of S. paradoxus and Z. mrakii and a lower abundance of A. oryzae, D. hansenii, N. crassa, Y. lipolytica, and U. maydis than subjects with normal weight (Fig. S10A at https://figshare.com/articles/figure/Supplementary_Figure_10_/21710141/2). In obese subjects, A. oryzae was slightly more abundant, and B. cinerea, Z. mrakii, and A. fumigatus had a negative log fold change compared to the normal group (Fig. S10B at https://figshare.com/articles/figure/Supplementary_Figure_10_/21710141/2). Underweight subjects differed from overweight in the abundance of Z. mrakii (positive log fold change), D. hansenii, A. oryzae, Y. lipolytica, N. crassa, S. paradoxus, and U. maydis (negative log fold change) (Fig. S10C at https://figshare.com/articles/figure/Supplementary_Figure_10_/21710141/2). Species exhibiting lower abundance were also similar in the case of a comparison of underweight versus obese subjects with the negative log fold change values for D. hansenii, Y. lipolytica, N. crassa, and U. maydis (Fig. S10D at https://figshare.com/articles/figure/Supplementary_Figure_10_/21710141/2). Additionally, three species, B. cinerea, S. paradoxus, and A. fumigatus, were detected in higher abundance in obese versus underweight subjects. Differences between obese and underweight subjects were the same as between obese and normal (Fig. S10E at https://figshare.com/articles/figure/Supplementary_Figure_10_/21710141/2).

### The effect of lifestyle on gut mycobiota.

**FIG 8:**
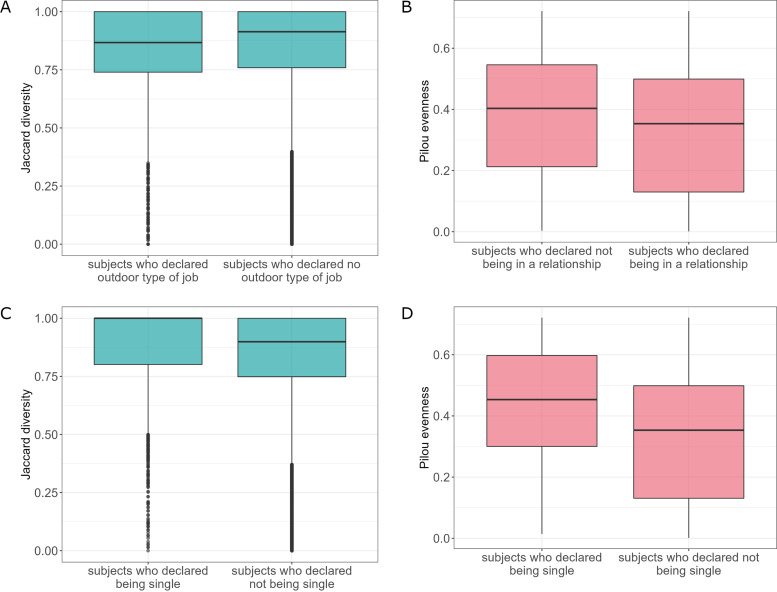
Characteristics of mycobiota taking into account lifestyle. (A) β-Diversities for the job outdoors factor (clr-transformed data). (B) Evenness of the analyzed community with respect to the marital status relationship factor. (C) β-Diversities for the marital status single factor (clr-transformed data). (D) Evenness of the analyzed community with respect to the marital status single factor.

Analysis of variance of the job outdoors factor showed statistically significant differences in β-diversity between tested groups (adonis, clr-transformed data, *P *= 0.004, *R*^2^ = 0.003) (Fig. 8A). However, differential abundance analysis of mycobiota was not able to reveal species that differ between tested groups within job outdoors participant data.

Subjects in a relationship exhibited less homogenous (or even) mycobial community than subjects who declared not being in a relationship (Pilou diversity, *P *= 0.015, Kruskal-Wallis rank-sum test) (Fig. 8B). Moreover, analysis of variance of the marital status single factor revealed statistically significant differences in β-diversity between tested groups (adonis, clr-transformed data, *P *= 0.015, *R*^2^ = 0.002) (Fig. 8C). Subjects who declared being single also had more homogenous mycobiota (Pilou diversity, *P *< 0.001, Kruskal-Wallis rank-sum test) (Fig. 8D), the opposite direction of changes than in the case of relationship. They also had a significantly lower abundance of S. cerevisiae than not-single subjects (Fig. S11 at https://figshare.com/articles/figure/Supplementary_Figure_11/21710147/1).

### The effect of health on gut mycobiota.

**FIG 9:**
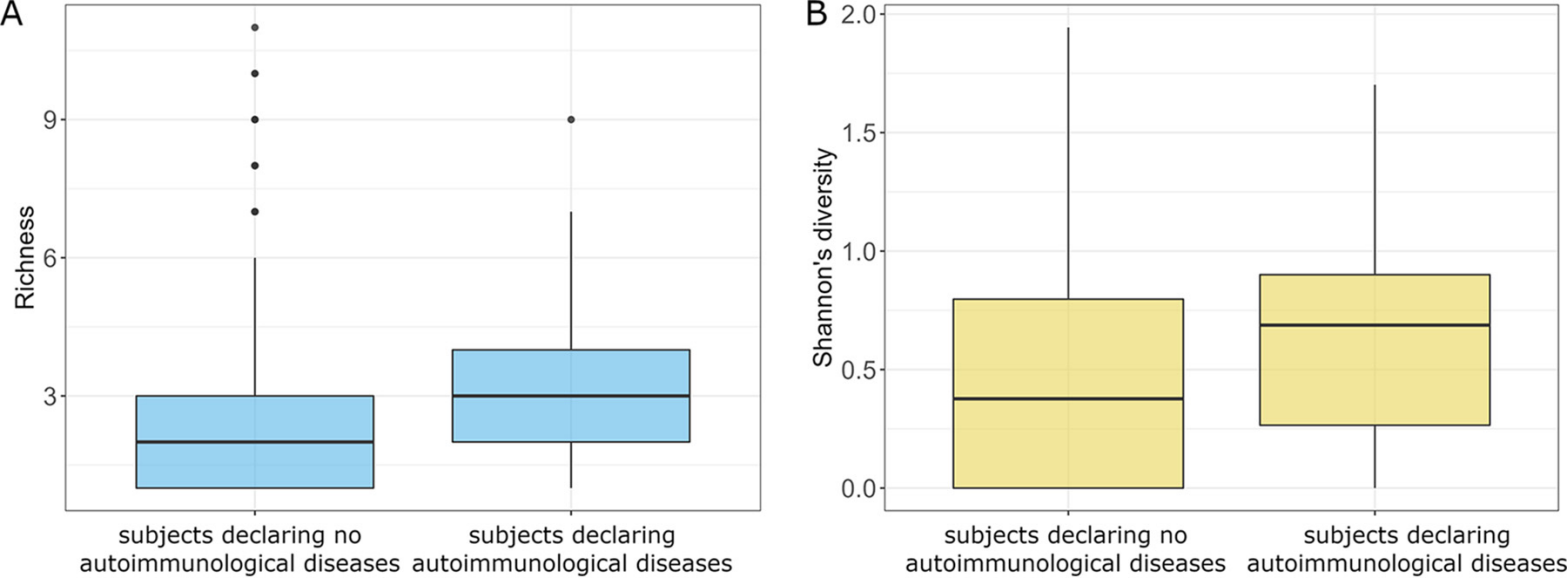
Comparison of subjects with and without autoimmunological diseases. (A) Richness of analyzed communities with respect to the autoimmunological diseases. (B) Shannon diversities of the analyzed communities with respect to the autoimmunological diseases.

Among the analyzed diseases, only the autoimmunological diseases affected the overall composition of the subjects’ mycobiome. Subjects with autoimmunological disorders had a higher richness of fungal species (Kruskal-Wallis rank-sum test, *P *= 0.020) (Fig. 9A) and higher Shannon diversity of gut mycobiota (Kruskal-Wallis rank-sum test, *P *= 0.005) (Fig. 9B). Moreover, subjects with autoimmunological diseases had a significantly lower abundance of D. hansenii (Fig. S12A at https://figshare.com/articles/figure/Supplementary_Figure_12/21710153/1).

Subtle changes in mycobiota were also detected by the analysis of variance in the case of reflux, irritable bowel, heartburn, and depression. Subjects with reflux had a lower abundance of S. paradoxus, N. crassa, and Z. mrakii (Fig. S12B at https://figshare.com/articles/figure/Supplementary_Figure_12/21710153/1). Abundances of N. crassa and Z. mrakii were also decreased in irritable bowel, accompanied by drops in C. dubliniensis, A. oryzae, M. restricta, and A. fumigatus (Fig. S12C at https://figshare.com/articles/figure/Supplementary_Figure_12/21710153/1). A lower abundance of A. oryzae was also detected in subjects with heartburn (Fig. S12D at https://figshare.com/articles/figure/Supplementary_Figure_12/21710153/1). Depression subjects showed diversified changes for several mycobiota species, including increases in N. crassa, B. cinerea, U. maydis, S. paradoxus, M. restricta, and A. oryzae and drops in Z. mrakii and A. fumigatus (Fig. S12E at https://figshare.com/articles/figure/Supplementary_Figure_12/21710153/1).

### The effect of medicines and supplements on gut mycobiota.

**FIG 10:**
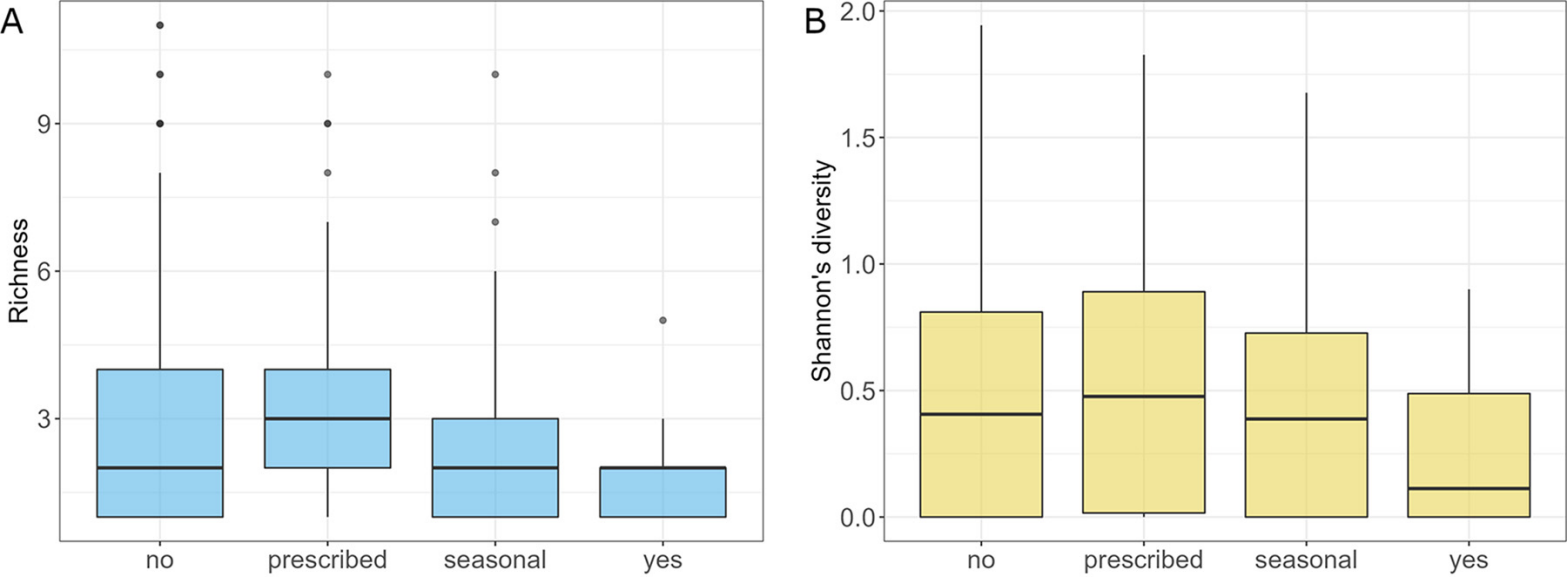
Comparison of subjects with respect to the probiotic uptake. (A) Richness of the analyzed groups. (B) Shannon diversities of the studied groups.

Probiotics affected the richness of gut fungal species (Kruskal-Wallis rank-sum test, *P *= 0.037). Subjects who took probiotics on a daily basis (a group declaring “yes”) had significantly lower richness and Shannon diversity of gut mycobiota than subjects who took probiotics only when prescribed (Conover's nonparametric all-pairs comparison test, *P *= 0.028 and *P *= 0.046) (Fig. 10A and B). Subjects who took probiotics on a daily basis had also significantly lower Shannon diversity of gut mycobiota than subjects who did not take probiotics (Conover's nonparametric all-pairs comparison, *P *= 0.048) (Fig. 10B). Analysis of variance between tested groups showed that people who took probiotics on a daily basis had a significantly higher abundance of S. paradoxus, N. crassa, C. dubliniensis, Y. lipolytica, B. cinerea, Z. mrakii, and A. fumigatus and lower abundance of U. maydis, M. restricta, S. graminicola, A. oryzae, and D. hansenii than subjects who did not declare taking probiotics (Fig. S13 at https://doi.org/10.6084/m9.figshare.21710159.v1). Contrary to probiotics, supplements did not influence mycobiota.

Surprisingly, different antibiotic uptake schemes did not affect fungal richness, Shannon diversity, Pilou diversity, and β-diversity. Despite that, differential abundance analysis showed that antibiotics may influence mycobiota structure (Fig. S14 at https://figshare.com/articles/figure/Supplementary_Figure_14/21710162/1). Subjects who declared no use of antibiotics had a higher abundance of M. restricta, N. crassa, U. maydis, and C. dubliniensis and a lower abundance of Z. mrakii, S. paradoxus, and A. oryzae than subjects who took antibiotics recently. Taking antibiotics more than a year ago manifested in a higher abundance of B. cinerea and M. restricta than recent antibiotic uptake. Subjects who took antibiotics 6 to 12 months before the examination also had a higher abundance of M. restricta but a lower abundance of Y. lipolytica and S. paradoxus. Comparison of mycobiota structure between subjects who took antibiotics recently and within half a year showed that people from the second group had a lower abundance of Z. mrakii, N. crassa, A. fumigatus, and A. oryzae.

Analyses of medications and supplement factors did not reveal differences at the level of fungal richness, Shannon diversity, Pilou diversity, and β-diversity. Also, the differential analysis did not detect species that differ between tested groups.

## DISCUSSION

This work characterizes the human gut mycobiota, not only in terms of taxa but most notably in a broad host-related context, including a wide range of factors, such as age, BMI, long-term habitual diet, health, and lifestyle. To our knowledge, this is the first large European cohort that was ever analyzed in terms of gut mycobiota associations with such a comprehensive and differentiated host-related set of factors. These data underscore the significance of the gut mycobiota in combination with population features (age, BMI, diet, lifestyle, etc.) for host health. Our extensive data set provides the essential characteristic of gut fungi composition in relation to lifestyle and host health that may be useful in dietary and clinical interventions. Host-related factors such as diet, age, and marital status exhibited substantial effects in mycobiota variations. Similar to other human-data-bacteriome association studies (20, 21), all of these statistically significant gut mycobiota covariates had a cumulative, nonredundant effect size of 14.54%. These data suggest that other, currently unknown elements and intrinsic microbial ecological factors substantially influence mycobiota variation. Although the effect of antibiotics was not significant, it exerted an effect size of 1.5% on population mycobiota variations.

### Core mycobiota.

Compared to bacterial communities, the human gut mycobiome is low in amount (~1% of all microbiota) and diversity (5). *Ascomycota* and *Basidiomycota* were the dominant phyla, in line with previous reports that also indicate the long-term stability of *Ascomycetes* (22, 23). Similar to other studies, gut mycobiota was dominated by yeast, including *Saccharomyces*, *Malassezia*, and *Candida* (22, 24). The prevalence of species belonging to *Saccharomyces* and *Candida* in the human gut is in line with earlier reports (6), whereas the *Sporisorium* genus was not previously described as a member of the human gut microbiota. Although intervolunteer variability in our cohort was high, and despite the fact that we do not track changes in intestinal mycobiota over time, the fact that several fungal species persisted across many samples, in conjunction with previous reports by other authors on the core mycobiome and the convergence of the results obtained in terms of frequently occurring species, may serve as a support for the hypothesis that a core gut mycobiota exists.

S. cerevisiae, C. albicans, and S. graminicola are the species that were found in a higher number of samples (58.79%, 31.61%, and 23.37%, respectively). Even though C. albicans and S. graminicola are present in less than half of the samples analyzed during the study, given the small number of fungal species detected in human fecal samples by whole-metagenomic approaches in general compared to the multitude of gut bacterial species and the composite nature of the metagenomic data, together with S. cerevisiae, they may seem to form the core fungal intestinal microbiota. Of notice, the reported frequency of particular fungal species may be also impacted by the indirect estimation of the abundance of species present in the intestine on the basis of fecal samples, which can lead to the underestimation of the abundance of fungal communities present in the inner surfaces of the intestinal mucosa (25).

Of these, S. cerevisiae can be widely found in the environment and food, indicating the potential origins or sources of the fungi in the human gut and reflecting that human health may be inherently affected by the environment and diet. On the other hand, C. albicans, which is a frequently detected fungus in feces of healthy humans, appears to have no major environmental reservoir, suggesting that it has extensively coevolved with its host and cohabiting microbes (2).

Of notice, S. graminicola, which is a plant pathogen, was not reported previously as a component of the human gut. This may be due to the particular dietary habits of the study cohort or the geographic occurrence of plants that are reservoirs of S. graminicola. In particular, the absence of S. graminicola in the results obtained by Sun et al. (4) can be explained by the differences between eating habitats of Chinese and European populations, as well as distinct geographic environments inhabited by these two populations. We also cannot exclude the possibility that S. graminicola was not correctly identified during our analysis or other analogous research due to limitations of used methods. In this context, it should be mentioned that Sun et al. (4) identified two other species belonging to the same genus as S. graminicola, namely, Sporisorium reilanum and Sporisorium scitamineum.

C. albicans can be considered an opportunistic pathogen. It is a normal component of the human gut microbiota (26, 27), but it can also colonize multiple other body sites (e.g., mouth, skin, vagina), where it commonly causes mucosal disease (28). Moreover, C. albicans can also disseminate from the human gut into the bloodstream and invade internal organs producing invasive, life-threatening infections (14, 29), although it should be noted that *Candida* infections typically occur in individuals with weakened immune systems, such as organ transplant recipients or cancer patients receiving chemotherapy (30), and in Europe, the incidence of invasive candidiasis is low (31).

### Diet and alcohol consumption.

As many fungi are used in the brewery and food industry, the impact of diet on human gut mycobiota appears likely. Studies suggest that this effect is broad and complex, especially for specific food categories (4, 6). The impact of the diet is 2-fold. On the one hand, many foods that contain fungi, such as yeast, can be a source of fungi in the gut. On the other hand, it is known that certain nutrients (e.g., sugars) can promote the growth of specific fungal species. Our research showed that diet-related factors, including alcohol consumption, are among the top significant covariates of mycobiota variation. Differences at the level of various diet-related factors also have the most profound and broad effect on the α-diversity of intestinal mycobiota. Differences between different groups were also reflected in β-diversity. Further, many fungal species were positively or negatively correlated with various diet-related factors, and many have statistically significant fold changes between groups.

The studies of the gut mycobiota suggest that the mycobiota variation is related to the composition of the recently consumed food (6), which is in contrast to the gut bacterial population variation for which previous studies emphasized that it was associated primarily with long-term diet (32, 33). In the previous study based on the Human Gut Microbiome Project (5), *Candida* was positively associated with diets high in carbohydrates but negatively with diets high in amino acids, protein, and fatty acids (6). Moreover, high *Candida* abundance was most strongly associated with the recent consumption of carbohydrates. The high prevalence of *Saccharomyces* was linked to the consumption of yeast-containing foods such as beer and bread (6).

### Host’s sex.

Although sex differences in microbiota composition are becoming evident (34, 35) both in animals and in humans, so far, studies have been mainly concentrated on bacteria, and little attention has been paid to the exact differences in the fungal composition between the sexes. Meanwhile, similar to bacteria, the differences in fungal composition between sexes can be attributed to the role of sex hormones in modulating microbiota composition (36) and diet in shifting the microbiota composition in a sex-dependent manner (37). Despite those assumptions, the only difference between males and females in fungal microbiota that we observed was at the level of homogeneity (Pilou index), with female subjects having more homogenous microbiota. In a study of the fungal composition of the intestinal tract performed by Strati et al. (7), measurements of the fungal richness within each analyzed sample based on the metagenomics analysis also showed no significant differences among male and female subjects. In contrast, the results based on the culture-based analysis from the previously mentioned study showed an increased number of gut fungal species in females compared to males. However, the authors did not identify significant differences between individual species amounts in males and females for any investigated age group in this study. They only observed that Aspergillus and some *Tremellomycetes*_unidentified_1 were significantly more abundant in male than female subjects. Also, contrary to our results, a greater diversity of fungal species was previously reported for women (38). Notably, this association only occurred in young but not middle-aged adults, and, as in our research, the subjects were mostly middle-aged. This could be the reason why we did not observe such differences between males and females.

### Age.

Association between age and microbiome structure are among the most studied topics regarding gut microbiota. Although the research indicates that the most evident change in gut microbiota diversity occurs in early childhood (39, 40), its increase in adulthood has also been reported (41, 42). Analysis of gut microbiota in different age groups showed a rise in α-diversity measures in young adults, with the trend halted at about 40 years (38). However, it has to be noted that this research was based on the 16S rRNA gene sequencing and therefore focused on the bacterial community. The previously described investigation of human mycobiota in relation to age showed that the richness of the gut mycobiota of infants (0 to 2 years old) and children (3 to 10 years old) was higher than adults (≥18 years old) (7). Our analysis revealed that also during adulthood, changes in mycobiota can be observed, as older adults (group 46 to 65) have higher fungal microbiota Shannon diversity than other investigated groups. Additionally, in our study, in general, the largest amount of differences in fungal species could be observed in comparison with the elderly. We also reported several changes at the individual fungal species level between all investigated age groups. These results show that age is an important factor that should be considered when investigating gut mycobiota.

### BMI.

Studies of lean and obese mice imply that the gut microbiota affects the energy balance, influencing the efficiency of calorie harvest and how this energy is used and stored (43). Obesity was correlated with a significant decrease in the level of bacterial diversity. Moreover, an increased *Candida* population was observed in obese individuals (6, 8, 44). The gut fungus Candida parapsilosis was recently identified as a critical commensal fungus related to diet-induced obesity in mice (45). Borges et al. suggested that obese individuals seem to be displaying higher yeast counts (8). This finding appears to be confirmed by our results for humans, as D. hansenii and S. paradoxus, which are yeast in the family Saccharomycetaceae, are among the species having the positive log fold change in individuals exceeding normal weight compared to underweight subjects. The high abundance of D. hansenii in the gut mycobiota may be related to the eating habits of people with obesity and overweight, as D. hansenii is a common species in all types of cheese (46) and sausages (47). However, at this point, it is still not clear whether an abnormal weight gain is induced by the specific fungi or, instead, changes in gut fungal mycobiota are the result of an energy-rich diet shift in obese and overweight individuals.

### Health.

Clinical studies show that the gut microbiome composition associates with human health (different diseases) (48, 49). Microbiome composition was associated with increased risks for certain diseases such as obesity, childhood allergies, diabetes, and inflammatory bowel disease and is altered in children with autism spectrum disorder (50–53). Within our study, among the analyzed conditions, only the autoimmunological diseases affected the overall characteristics of the subjects’ mycobiota, with subjects with autoimmunological disorders having a higher richness and Shannon diversity of intestinal mycobiota. Interestingly, subjects with autoimmunological diseases had a significantly lower abundance of D. hansenii. So far, the role of D. hansenii in human health is not clear. In the context of autoimmunology, D. hansenii was recently linked to Crohn’s disease, where it prevents intestinal healing (13), and, earlier, with an impact on rheumatoid arthritis in rats (54). The present research provides hints at the influence of D. hansenii on autoimmunological diseases, although further studies are required to investigate this relationship more deeply.

We also detected a drop in several species’ abundances in digestive disorders such as reflux, irritable bowel, and heartburn, namely, S. paradoxus, N. crassa, Z. mrakii, C. dubliniensis, A. oryzae, M. restricta, and A. fumigatus. These results might seem contradictory, as it is widely accepted that mycotoxins generated as fungal metabolites contribute to disturbances of gastrointestinal barrier and immune functions and are therefore associated with digestive problems (55). However, taking into account that some species such as S. cerevisiae can also positively impact human health and even act as probiotics (56, 57), it cannot be ruled out that identified species can also exhibit positive effects on the human intestinal tract. The drop in their abundance may therefore promote digestive problems.

The most diversified pattern of changes at the level of individual fungal species was observed for depression, with increases in N. crassa, B. cinerea, U. maydis, S. paradoxus, M. restricta, and A. oryzae and decreases in Z. mrakii, and A. fumigatus. The impact of gut microbiota on mental health, including depression, attracted significant attention over the past decade, and several mechanisms of bidirectional influence between gut microbiota and mental health were proposed (58). However, research was largely focused on a bacterial part of the gut microbiota, and even though there is some evidence of a plausible gut mycobiota-brain axis, we still lack an understanding of the underlying mechanisms by which gut mycobiota affects the brain.

Regarding the mycobiome and depression, Jiang et al. studied the microbiota of patients with current depressive episode (CDE) (59). They showed that gut mycobiota of CDE patients was characterized by a relative reduction in α-diversity assessed using the ACE and Chao indices, whereas, similar to our results, the Shannon index-based α-diversity and β-diversity were not significantly different between CDE and healthy groups. Moreover, the CDE group had higher levels of *Candida* and lower levels of *Penicillium* than the control group. Our research is the first that shows the diversified pattern of changes in gut fungal microbiota at the species level in depression. The findings of Jiang et al. concerning the changes at the genus level cannot be directly compared to our results, as the two analyses were performed at different levels, genus versus species levels. For instance, in the case of our analysis, two fungal species A. fumigatus and A. oryzae, belonging to the same Aspergillus genus, were detected as having an opposite pattern of changes in subjects declaring depression (although log fold changes for these species were very small). Furthermore, Jiang et al. (59) showed that a gut microbial index based on the combination of eight genera (four bacterial and four fungal CDE-associated genera) distinguished CDE patients from controls, suggesting that the gut microbiome signature is a promising tool for disease classification. Of notice, both Jiang et al. and our studies are observational, and the numbers of samples belonging to patients with depression are small (24 and 30, respectively). In order to determine the role of gut mycobiome in depression, future metagenomic studies involving larger cohorts accompanied by metabolomics analyses are needed.

### The use of medicines.

Antibiotics have a well-documented detrimental effect on gut bacterial composition (60–63), but their impact on the fungal community is less apparent and was not studied widely. Theoretically, commensal bacteria may limit fungal colonization and invasion by several mechanisms, such as the production of antifungal compounds (64), competition for available nutrients, chemotaxis, and physiochemical changes to the local environment (65, 66). Therefore, drugs targeting bacteria can be an essential risk factor for fungal infections. In a study of the impact of antibiotics on the gut mycobiota, it was demonstrated that antibiotic administration induced significant changes in gut bacteria that translated into long-term changes in fungal abundance (16). While bacterial communities recovered mostly 30 days after antibacterial treatment, the fungal community has shifted from mutualism to competition. However, it should be emphasized that the study size was small, as only 14 participants were analyzed. Most studies up to date concentrated on pathogenic fungi. It has been shown that antibiotics specific to anaerobic bacteria or broad-spectrum antibiotics can have differential impacts on fungal susceptibility, particularly C. albicans (17, 67). Another study demonstrated that commensal fungi such as C. albicans or S. cerevisiae can functionally replace intestinal bacteria in bacterial dysbiosis after the exposition to an antibiotic (18). Patients with antibiotic-associated diarrhea have *Candida* overgrowth in the gastrointestinal tract (19). On the other hand, diet therapy and antibiotics seem to reduce the abundance of fungal species in patients with Crohn’s disease (68).

In our study, we did not observe global changes in the gut mycobiota composition in terms of α- and β-diversities. However, at the level of individual species, alterations were visible. Recent uptake of antibiotics was associated with a higher incidence of Z. mrakii, A. fumigatus, and A. oryzae. A. fumigatus is a pathogenic fungus, so its higher incidence after antibiotic treatment is undesirable. This is especially dangerous for immunodeficient subjects, as A. fumigatus is the most frequent cause of invasive fungal infection in immunosuppressed individuals (15, 69, 70).

### Limitations.

The main limitation of our study lies in its descriptive nature and the fact that associations are not proof of causation. Some of our conclusions are speculative and correlative, and therefore, future studies are needed to discover the cause-and-effect relationship between the gut mycobiome and host factors, as well as the downstream mechanistic aspects. Moreover, only single samples were taken from individuals, and we did not track changes in gut mycobiome over time. Therefore, determination of how representative observed fungal communities are in people's gut across time requires further study. Also, an estimation of the abundance of particular fungal species in gut was indirect, as it was based on the fecal samples that do not fully represent the mucosal-associated fungal communities and that can differ in biological significance (25).

Nevertheless, despite all the limitations, multiomic analyses of large cohorts supported by extensive human data can significantly improve our understanding of the ecology of the gut mycobiota in the context of host-related factors. Thanks to this, they can quickly suggest potentially valuable paths for further narrowly focused research on gut mycobiome and its impact on human health.

### Conclusions.

Compared to bacterial communities, the human gut mycobiome is low in amount (~1% of all microbiota) and diversity and dominated by yeast, including *Saccharomyces*, *Malassezia*, and *Candida*. Although intervolunteer variability in the investigated cohort was high, several fungal species persisted across most samples, which is evidence that a core gut mycobiota exists. Moreover, we showed that host-related factors such as diet, age, and marital status influence the variability of gut mycobiota and, therefore, human health. To our knowledge, this is the first large European cohort that was analyzed in terms of gut mycobiota associations with such comprehensive and differentiated host-related factors. Moreover, apart from the global differences at the level of α- and β-diversity of gut intestinal mycobiota, our analysis revealed changes in abundances at the fungal species level for many investigated factors. In the coming era of the gut microbiome in precision medicine, further research into the functional consequences of different mycobial structures deserves in-depth investigation.

## MATERIALS AND METHODS

### Cohort description and study participants.

Stool samples were collected from 923 participants and are a part of the Polish Microbiome Map project (ClinicalTrials.gov study identifier NCT04169867). Informed consent was obtained from all the participants for the collection of stool samples and study information. Each participant completed a detailed medical, lifestyle, and dietary questionnaire. Participants were broadly representative of the overall Polish adult (>18 years old) population with respect to age, health, education, and marital status. Human data are available in Table S1 in the supplemental material. Samples were taken from 2020 to 2021.

### Metagenomic sequencing.

The sampling, DNA isolation, high-throughput sequencing library preparation, and sequencing were performed according to the protocol elaborated on and described in our previous studies (71, 72).

### (i) Sampling and storing volunteer samples.

Fecal samples (approximately 1 g) were self-collected by all donors into vials containing 3 mL of RNAlater stabilization solution (Invitrogen, Thermo Fisher Scientific) and delivered by courier within 24 h to the laboratory, where the samples were anonymized and stored at 4°C for up to 1 week. Each person provided signed informed consent for participating in the study. Appropriate approval was also obtained from the Bioethical Commission of the Karol Marcinkowski University of Medical Sciences, Poznan, Poland (no. 316/22, passed on 14 April 2022, as an extension of resolution no. 485/19, passed on 11 April 2019). Each sample was homogenized. Tubes were centrifuged at 14,000 × *g* for 5 min, the supernatant was discarded, and residues were transferred to a −20°C freezer for storage until DNA extraction (usually a few weeks).

### (ii) DNA isolation.

The frozen stool samples were thawed on ice, and DNA was extracted from them using a DNAeasy PowerSoil Pro kit (Qiagen, Germany) according to the manufacturer’s instructions. The following protocol adjustments were applied. The liquid phase of stabilized stool samples was thoroughly discarded to remove high salt content that may interfere with a subsequent DNA purification step. Next, the stabilized stool samples (250 mg) were bead-beaten in PowerBead Pro tubes containing proprietary beads using a Mixer Mill MM400 (Retsch, Germany) for 15 min at 25 Hz. To remove RNA and increase DNA yield, each sample was incubated with 5 μL RNase (10 mg/mL concentration; A&A Biotechnology, Poland) at 60°C for 10 min. The DNA quality was verified with agarose gel electrophoresis. The final DNA concentration was measured by a NanoDrop ND-1000 spectrophotometer (Thermo Fisher Scientific, USA).

### (iii) DNA library preparation.

Libraries were constructed with the TruePrep DNA library prep kit V2 for Illumina TD501 (Vazyme Biotech, China) according to the manufacturer’s protocol. We used 500 ng of stool-extracted DNA for each library preparation. In the library amplification step, six PCR cycles were applied. Library concentration was measured using a Qubit fluorometer and Qubit DNA high-sensitivity (HS) assay kit (Thermo Fisher Scientific, USA). The quality of libraries and fragment distribution were analyzed using a Bioanalyzer 2100 and DNA 1000 kit or high-sensitivity DNA kit (Agilent Technologies, USA), depending on the obtained library quantity.

Purified libraries were stored for a few weeks at −20°C until sequencing.

### (iv) High-throughput sequencing.

Before sequencing, all libraries were thawed on ice and normalized to the final 10-nM concentration. Libraries with distinctive index combinations were pooled and diluted with EB buffer (Qiagen, Germany) to obtain a mix of 2-nM libraries, according to Protocol A: Standard Normalization Method for the NextSeq System (Illumina, USA). Sequencing was performed with NextSeq 550 (Illumina, USA) using high-output kit v2.5 reagents (Illumina, USA); approximately 10 million 150-bp paired-end reads were generated per library. Neither human DNA sequence depletion nor enrichment of microbial or viral DNA was performed.

### (v) Data preprocessing and quality control.

Demultiplexing was run on the raw BCL intensity file with the bcl2fastq tool (73) for base calling and separating the reads from different samples. To assess the quality of the sequencing procedure, we generated quality control reports with FastQC (74) and MultiQC (75). We preprocessed the raw FASTQ reads with cutadapt (76) using the following procedure: we trimmed the adapter sequences (based on TruSeq adapter sequences) and poly(G) tails observed in the data, which are characteristic of the two-channel sequencing technology of NextSeq. We also filtered out reads shorter than 140 bases to remove the bias in taxonomy profiling that could emerge from the shorter sequences. The remaining reads were subjected to further analysis.

### Bioinformatic analysis.

**(i) Fungal community profiling.** Profiling of the fungal microbiome was performed via Kraken2 (77), followed by Bracken (78). Kraken2 is a classification system that uses exact matches of the k-mers from the query sequence to the lowest common ancestor of all the genomes in the database holding this k-mer to inform the classification algorithm. Bracken is an accompanying tool of Kraken2 that is used to obtain a quantitative profile of the samples. Bracken employs probabilistic reestimation of taxa abundance based on Kraken’s read-level taxonomy assignment. For the taxonomy assignment with Kraken2, we used a complete Kraken2 database provided by the tool developers.

**(ii) Statistical analysis.** To assess how the level of sequencing depth relates to the observed richness across samples, rarefaction curves for the detected species were calculated for the whole data set, including all kingdoms of life (rarefy and rarecurve functions, R package vegan) (Fig. S15 at https://figshare.com/articles/figure/upplementary_Figure_15/21710165/2).

For α-diversity, the following metrics were calculated: richness, evenness (Pilou evenness), and effective diversity (Shannon’s index, diversity function, R package vegan) (79, 80). Fungal richness (species count) was calculated by counting the number of species detected at least once in a given sample. Pilou evenness was calculated by dividing the diversity (Simpson’s index, diversity function, R package vegan) by log(*S*), where *S* is the total number of species. To analyze the structure of mycobial communities across samples (β-diversity), data were log-ratio centered (clr transformation, clr function, compositions R package), and the principal-component analysis (PCA, Jaccard distance matrix, R package vegan) was employed. Additionally, principal-coordinate analysis (PcoA; Aitchison distance, R package vegan) of samples was performed to illustrate how the community structures of individual samples across the study population differ (Fig. S16 at https://figshare.com/articles/figure/Supplementary_Figure_16/21710171/1). A pseudocount of 0.005 was added to the species raw count prior to distance calculation to avoid zeros, as clr transformation cannot be used with nonpositive data.

Statistical testing of α-diversity was performed using the Kruskal-Wallis test. Multiple-pairwise comparison between groups was performed with the Conover's nonparametric all-pairs comparison test (kwAllPairsConoverTest function, PMCMRplus R package, false-discovery rate [FDR] < 5% [81]). For β-diversity significance testing, permutational multivariate analysis of variance using distance matrices was conducted (adonis [82], Jaccard method, vegan R package, 999 permutations, FDR < 5%), accompanied by the Wilcoxon rank-sum test with continuity correction.

To identify fungal species contributing to community compositional dissimilarities, the analysis of compositions of microbiomes with bias correction was performed (ANCOM-BC; ANCOMBC Bioconductor R package [83]).

Covariates of mycobiota variation were identified by calculating the association between continuous or categorical phenotypes and species-level community ordination (PCA) with the envfit function in the vegan R package (999 permutations, FDR < 5%). This function performs multivariate analysis of variance (MANOVA) and linear correlations for categorical and continuous variables, respectively.

Associations between fungal genera and investigated factors were calculated with Cramer V correlation coefficient estimation with bias correction (cramerV function from rcompanion R package), and their significance was estimated with the chi-square test with continuity correction (chisq.test function from stats R package). For the statistical significance calculation, only genera identified in at least five samples were taken into account.

Co-occurrences of fungal species in the samples were calculated as cross-products of the individual species’ pairs detected in samples (crossprod function, R). Data clustering was performed via hierarchical clustering with Euclidean distance.

Correlations between fungal species were calculated with the corr.test function from the psych R package (84). Data were log-ratio centered (clr transformation, clr function, compositions R package). Pairwise Spearman correlations were used, and their significance was estimated with the *t* test with FDR adjustment; a *P* value of 0.05 was used for confidence intervals. Two-tailed probability of *t* test for each correlation was used as an indicator of significance.

In this article, we present only statistically significant results.

### Ethics approval and consent to participate.

Patients and the public were not involved in the design, conduct, reporting, or dissemination plans of our research.

This study involved human participants and was approved by the Bioethical Commission of the Karol Marcinkowski University of Medical Sciences, Poznan, Poland (resolution no. 316/22, passed on 14 April 2022, as an extension of resolution no. 485/19, passed on 11 April 2019). Participants gave informed consent to participate in the study before taking part. All methods were carried out in accordance with relevant guidelines and regulations.

### Data availability.

Data are available in a public, open-access repository. The raw data are available in the NCBI SRA repository (https://www.ncbi.nlm.nih.gov/sra) under BioProject accession number PRJNA850529. Other data sets generated during and/or analyzed during the current study are available from the corresponding author upon reasonable request. STORMS checklist for the study is available in Table S3 in the supplemental material.

10.1128/msystems.00986-22.3 TABLE S3 Strengthening The Organization and Reporting of Microbiome Studies (STORMS) checklist for the study. Download Table S3, XLSX file, 0.07 MB. Copyright © 2023 Szóstak et al. 2023 Szóstak et al. https://creativecommons.org/licenses/by/4.0/ This content is distributed under the terms of the Creative Commons Attribution 4.0 International license.
